# Towards quantitative assessment of cerebrovascular autoregulation in human neonates using ultrafast ultrasound imaging

**DOI:** 10.1038/s41598-025-97292-w

**Published:** 2025-04-11

**Authors:** Nikan Fakhari, Julien Aguet, Alison Howell, Minh Nguyen, Luc Mertens, Lynn Crawford, Maelys Venet, Christoph Haller, David Barron, John G. Sled, Jérôme Baranger, Olivier Villemain

**Affiliations:** 1 Department of Medical Biophysics, University of Toronto, Toronto, ON Canada; 2 Department of Pediatrics, Division of Cardiology, The Hospital for Sick Children, Toronto, ON Canada; 3 Department of Diagnostic and Interventional Radiology, The Hospital for Sick Children, Toronto, ON Canada; 4 Division of Cardiology, Texas Children’s Hospital, Baylor College of Medicine, Houston, TX USA; 5 Department of Anesthesia and Pain Medicine, The Hospital for Sick Children, Toronto, ON Canada; 6 Department of Pediatric and Adult Congenital Cardiology, Bordeaux University Hospital (CHU), Pessac, France; 7 Department of Surgery, Division of Cardiovascular Surgery, The Hospital for Sick Children, Toronto, ON Canada; 8 Department of Surgery, University of Toronto, Toronto, ON Canada; 9 Mouse Imaging Centre, The Hospital for Sick Children, Toronto, ON Canada; 10 Department of Translational Medicine, The Hospital for Sick Children research institute, Toronto, ON Canada; 11 Physics for Medicine Paris, INSERM U1273, ESPCI Paris, CNRS FRE, PSL Research University, Paris, France

**Keywords:** Cerebrovascular autoregulation, Neonates, Pediatrics, Cardiac surgery, Ultrafast ultrasound imaging, Physiology, Neurophysiology

## Abstract

Newborns with congenital heart diseases requiring cardiopulmonary bypass (CPB) are at risk of neurodevelopmental impairment. The impact of deep hypothermia cardiopulmonary bypass (DH-CPB) on cerebrovascular autoregulation (CAR) that controls brain perfusion in the presence of blood pressure variation is not well understood. Recently, ultrafast power Doppler (UPD) showed potential to study CAR in neonates based on cerebral blood volume (CBV). However, since CAR relies mainly on arterial vasoconstriction/vasodilation, monitoring of brain perfusion variation based on CBV requires the discrimination of arterial from venous CBV. This study aims to use UPD combined with an algorithm for the discrimination of arteries and veins to monitor CAR during DH-CPB in neonates. Transfontanellar ultrafast power Doppler was performed in two groups of newborns: those undergoing deep hypothermic cardiopulmonary bypass with circulatory arrest (18–20 °C, *n* = 6, “DH group”) and those undergoing full-flow CPB at mild hypothermia (32–34 °C, *n* = 6, “non-DH group”). Blood flow directionality was used to differentiate arterial compartments of CBV from venous CBV in specific brain regions where arterial and venous flows exhibit opposite directions. To study CAR, a linear mixed effect model was used to find the association between arterial CBV and mean arterial blood pressure (MAP). In the “non-DH group”, we found a negative association between arterial CBV and MAP, indicating that an increase in MAP is associated with a decrease in arterial CBV (slope = -0.020 $\:{mmHg}^{-1}$, *p* = 0.047). Conversely, in the “DH group” no significant association was found such that arterial CBV remained stable as MAP increased (*p* = 0.314). We interpret the reduction in arterial CBV with increasing MAP in the “non-DH group” as an active arterial vasoconstriction triggered by CAR, whereas the lack of variation of arterial CBV in the DH group suggests impaired CAR response. Our findings highlight the potential of ultrafast ultrasound imaging for intra-operative CAR monitoring, paving the way for a better understanding of the impact of different types of CPB on cerebral perfusion.

## Introduction

Approximately 50% of patients diagnosed with critical congenital heart disease develop variable degrees of neurodevelopmental impairment^1^. Neonatal cardiac surgery involving cardiopulmonary bypass (CPB) poses additional risks to neurodevelopment outcomes^2^. However, the extent to which preoperative and intra-operative factors contribute to neurological injury is unknown. A potential mediator of neurological injury is impaired cerebrovascular autoregulation (CAR), which is common before, and during cardiac surgery requiring CPB^3–6^. CAR is a protective mechanism of the brain that maintains constant cerebral blood flow during changes in arterial blood pressure by modifying the cerebrovascular tone to allow vasodilation or vasoconstriction^7^. This autoregulation occurs within a range of blood pressure (lower and upper limits) in which cerebral blood flow exhibits a plateau. Growing evidence indicates that brain immaturity during the prenatal and postnatal preoperative periods may compromise the appropriate regulation of cerebral blood flow, thereby elevating the risk of brain injury^8^. Periventricular white matter injury is the most common MRI finding in the preoperative periods and it thought to results from higher susceptibility of immature cells to the preoperative ischemic insults and reduced cerebral blood flow^9^. Moreover, cardiac defects can lead to altered oxygen saturation due to intracardiac and extracardiac mixing, resulting in varying degrees of chronic hypoxia^1^. It is well documented that hypoxia can shift the lower limits of pressure that maintain a plateau on the autoregulation curve and alter regional cerebral blood flow in the developing brain^10^. Finally, specific surgical interventions, in particular, the use of deep hypothermia during cardiopulmonary bypass (DH-CPB) can adversely affect the CAR mechanism^11^, but the extent of these effects in children with congenital heart disease is not well understood. Therefore, developing robust techniques to monitor CAR and optimize neurovascular management strategies is crucial for neonates with congenital heart disease.

To date, several techniques in clinical practice allow for assessment of CAR. Intracranial pressure monitoring (ICP) is frequently used to study vasoreactivity in adults and children, for example^10^. Furthermore, indicator dilution techniques, ^133^Xenon clearance, as well as positron emission tomography provide direct measurements of cerebral blood flow, which are then used to study the pressure-flow relationship^12^. Unfortunately, these techniques have limited applicability in neonates and infants due to their invasive nature and exposure to ionizing radiation. Because of these constraints, several non-invasive and radiation-free techniques have been proposed. For instance, transcranial Doppler ultrasound enables CAR assessment by targeting major intracranial arteries (e.g., middle cerebral artery) and monitoring blood velocity variations as a surrogate for cerebral blood flow^13^. However, velocity measurements are highly angle-dependent, and this approach suffers from low spatial and temporal resolution and cannot provide information about the microvasculature where CAR is predominantly active^14^. Laser Doppler flowmetry represents an alternative approach that enables measurement of microvascular blood flow velocity, but the technique is limited by signal processing issues, significant motion artifacts, calibration challenges, and strong dependence on probe pressure that can affect the underlying blood flow measurements^15^. Near-Infrared spectroscopy (NIRS) is the most commonly used technique in neonates to assess CAR through longitudinal monitoring of cerebral tissue oxygen saturation and cerebral oximetry index. However, NIRS suffers from limited penetration depth and cannot assess autoregulation in deeper brain areas such as periventricular white matter, basal ganglia, and thalami, which are known to be more susceptible to brain injury^16^. Finally, non-invasive monitoring of ICP recently proposed by Hassett et al.^17^ uses a mechanical extensometer device that detects skull deformations from pulsatile ICP variations to calculate the pulse amplitude index as a measure of cerebrovascular reactivity. However, this approach is highly sensitive to strong motion artifacts.

A potential alternative strategy to overcome these limitations is to utilize ultrafast ultrasound power Doppler (UPD). Recently, UPD was applied in neonates during DH-CPB and revealed its potential to study CAR based on the measurement of total cerebral blood volume (CBV)^18^. Currently, a significant challenge in UPD-based CAR assessment lies in the non-specific nature of CBV, encompassing both arterial and venous blood volumes. Arterial vessels contain robust, smooth muscle layers, enabling active regulation of vessel diameter in response to systemic blood pressure changes^19^.

Consequently, in scenarios where arterial blood pressure decreases or increases, one would expect a corresponding increase or decrease in arterial CBV due to vasodilation or vasoconstriction, respectively^20^. In contrast, venous vessels primarily exhibit passive compliance to changes in arterial blood pressure; thus, changes in arterial pressure are not anticipated to affect venous CBV when autoregulation is intact. Hence, the separation of arterial and venous compartments of CBV is crucial for the comprehensive understanding of CAR. Using the advantages of ultrafast ultrasound imaging, this distinction can be achieved^21^. Therefore, this study aims to assess UPD combined with arteries and veins discrimination for quantitative assessment of CAR in neonates during DH-CPB.

## Materials and methods

### Study design (See Fig. 1)

**Fig. 1:**
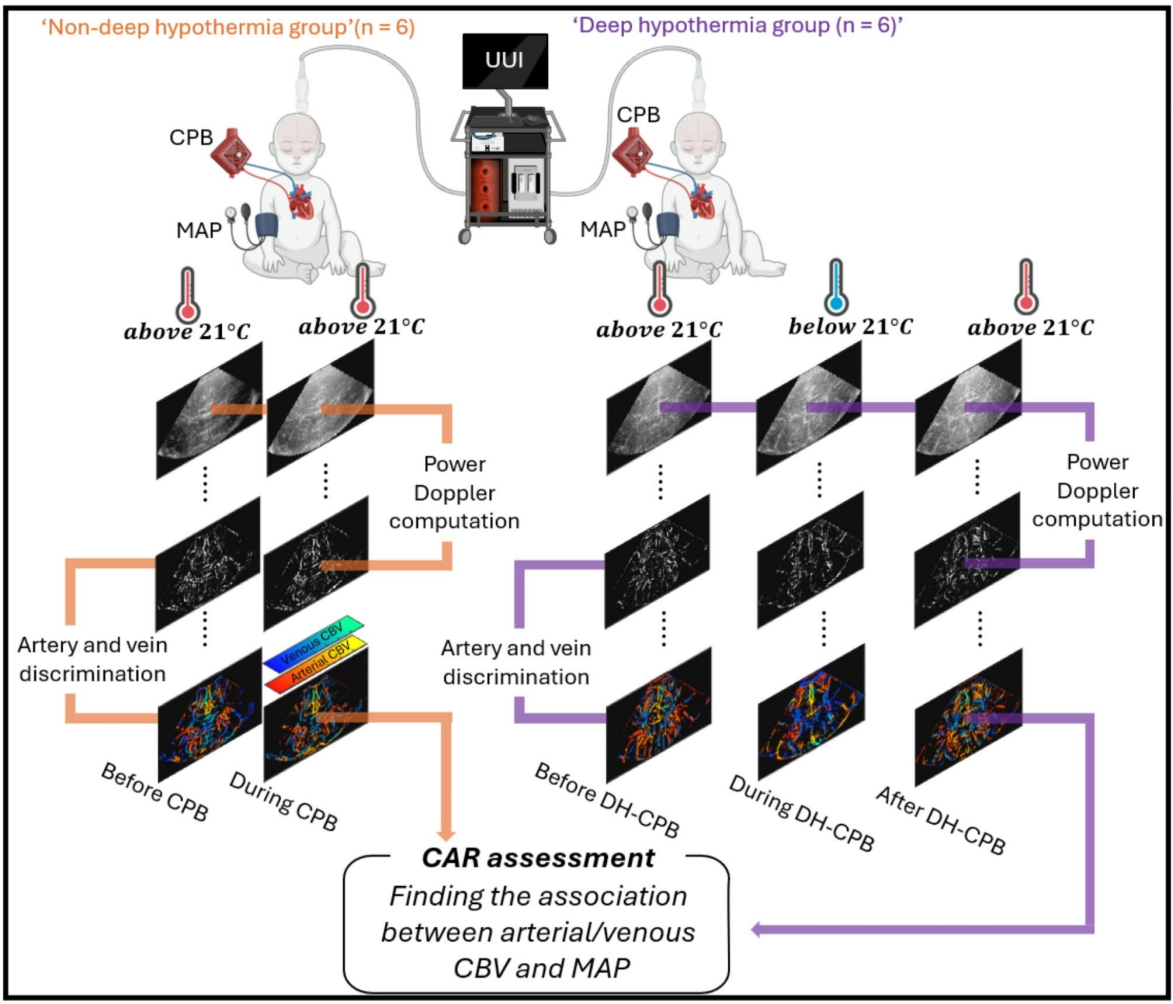
Study Design. CBV = cerebral blood volume; CAR = cerebrovascular autoregulation; DH-CPB = deep hypothermic cardiopulmonary bypass; MAP = mean arterial blood pressure; UUI = ultrafast ultrasound imaging.

This was an observational pilot study exploring a novel imaging technology implementation. The study was approved by the Institutional Review Ethics Board (#1000073382) at The Hospital for Sick Children in Toronto, Canada. Patients were consecutively recruited from our cardiac surgery program at this single center. Informed consent was obtained from the parents or legal representatives. All methods were performed in accordance with the relevant guidelines and regulations. The study included two groups: (1) newborns with hypoplastic left heart syndrome (HLHS) undergoing CPB at deep hypothermia with circulatory arrest (18–20 °C, *n* = 6, “DH group”) and (2) newborns with transposition of the great arteries undergoing full-flow CPB at mild hypothermia (32–34 °C, *n* = 6, “non-DH group”). Of the initial 9 patients enrolled in each group, 3 were excluded due to inadequate image quality, resulting in 6 patients per group who completed all planned ultrasound scans. We must clarify that for this study we did not perform any sample size calculation as this was designed as a pilot, proof-of-concept study aimed at establishing the feasibility of the imaging protocol and providing preliminary comparative data between the two temperature management strategies. The inclusion criteria included neonates undergoing cardiac surgery with cardiopulmonary bypass, while exclusion criteria included any contraindications to ultrasound imaging and inability to obtain parental consent. All participants underwent a pre-operative brain MRI scan, which showed no structural abnormalities. MRI is part of the standard of care for these patients with predefined and standardized protocols which is comparable for every participant. Moreover, no signs of syndromic conditions were identified during pre-operative clinical assessment, as confirmed by our physicians. The “non-DH group” underwent the arterial switch operation, and the “DH-group” underwent the Norwood-Sano operation. We must clarify that the Norwood Sano operation involves complete reconstruction of the aortic arch, necessitating a period during which perfusion to the head and neck vessels is interrupted or compromised. To protect the brain during this phase, deep hypothermia is routinely employed. In contrast, the arterial switch operation focuses on the aortic root, eliminating the need for circulatory arrest or deep hypothermia. The anesthesia strategy was similar for both patient groups (isoflurane). Initial CPB flow rates for each group were determined by institutional practice with minimum target ranges between 100 and 120 ml/kg/min. Intraoperative clinical data were recorded, including surgery time (defined as the period from anesthesia initiation to completion), continuous monitoring of partial pressure of carbon dioxide (PCO_2_), and mean arterial blood pressure (MAP). Cerebral venous pressure is typically low (4–8 mmHg)^22^ and not easily measured, particularly in small, sick infants^23^. Hence, in this study, MAP was used as a surrogate of cerebral perfusion pressure for CAR assessment. The timing of ultrafast ultrasound scans was synchronized with continuous MAP measurements to ensure accurate temporal correlation. During CPB, MAP was primarily managed for both groups through pump flow rate adjustments, maintaining target ranges of 150–200 mL/kg/min. When additional MAP support was required, phenylephrine boluses were administered, and hematocrit was optimized through transfusion as needed. In cases requiring sustained pressure support, norepinephrine infusion was initiated. For elevated MAP, interventions included reduction of pump flow rate while maintaining minimum thresholds (100–120 mL/kg/min), increasing isoflurane concentration, or initiating sodium nitroprusside infusion. All pressure management strategies were implemented with verification of proper arterial cannula positioning.

### Ultrafast ultrasound acquisitions

For the “DH-group”, intra-operative ultrafast ultrasound scans were performed at three different time points: before, during, and after DH-CPB. For each of these time points, two ultrafast ultrasound scans were acquired. The scans acquired before DH-CPB were (1) after the induction of anesthesia and (2) after CPB started. The scans acquired during DH-CPB were (1) during selective cerebral perfusion and (2) during aortic arch reconstruction. Lastly, the scans acquired after DH-CPB were (1) after unclamping the aorta and (2) post-CPB (during rewarming). For the “non-DH group”, two ultrafast ultrasound scans were acquired. The first one was upon Induction of anesthesia, and the second one was after CPB started, as shown in Fig. 1. The ultrafast ultrasound scans were performed in the coronal plane utilizing a Verasonics Vantage 256 research system (Verasonics, USA) with a phased array probe (GE 6 S-D, 5.7 MHz, GE Healthcare, USA)^24^. A trained pediatric radiologist (J.A.) precisely identified the coronal plane using conventional real-time B-mode at specific anatomical landmarks, including the foramina of Monro, basal ganglia, and pons, before ultrafast acquisitions^18,25,26^. Once the desired plane was established, the ultrafast ultrasound sequence was performed. This sequence comprised 5 compounded diverging waves (with a sector width of 90 degrees) coherently compounded following the method outlined by Papadacci et al.^27^ with a pulse repetition frequency of 9 kHz and a duration of 0.67 s.

### Ultrafast power doppler computation

From the obtained ultrafast sequences, beamforming was performed using the delay.

and sum approach^28^. In the next step, an adaptive spatiotemporal clutter-filter, using singular value decomposition was applied to the beamformed data to separate the blood signal from the tissue signal^29^. This cluttered-filtered ultrasound data was then time-averaged, and scan converted to generate a UPD Image^24^.

### Separation of cerebral arteries and veins

In prior work, we introduced ultrafast Doppler estimators to differentiate between cerebral arteries and veins in healthy newborns^21^. Our findings indicated that normalized ultrafast Doppler spectrogram (NDS) achieved the highest classification performance across various regions of the brain (AUC = 0.92). The artery and vein classification based on NDS relies on characteristic spectral differences between arterial and venous flow: arteries exhibit a pulsatile flow, while veins have a continuous flow. Unfortunately, during CPB, this technique cannot be applied due to the non-pulsatile flow generated by the CPB pump. Acknowledging this technical challenge, we opted to use the second-best estimator for artery and vein classification in the current study: the directivity of blood flow (AUC = 0.87). It is important to note that the robustness of blood flow directivity as an estimator is inconsistent across various brain regions. For instance, this estimator has poor classifying performance at the level of the cingulate gyrus (CG), where the direction of both arterial and venous flow varies greatly between vessels. Therefore, we excluded this region from all quantitative analyses for this study.

In this study to discriminate between arteries and veins the following steps were used: First, a signed-UPD image representing the blood flow directionality towards or away from the ultrasound transducer was obtained following the methodology described in Mace et al.^30^. Subsequently, arteries and veins were discriminated based on the opposite arterial/venous flow directivity in the following regions of interest: (1) cortico-subcortical areas, (2) basal ganglia and (3) remaining brain structures. Here, the remaining brain structures are defined as the brain accessible area (e.g., full imaging filed of view) minus the surface area covering basal ganglia, cortico-subcortical areas, and the CG area. The following arterial and venous flow directionalities were utilized: within cortico-subcortical areas; upward flow indicated venous flow, whereas downward flow indicated arterial flow. In basal ganglia, upward flow indicates arterial flow, whereas downward flow indicates venous flow, and in remaining brain structures, upward flow indicates arterial flow, whereas downward flow indicates venous flow.

### Computation of arterial and venous CBV

Once arteries and veins were discriminated, arterial and venous CBV were computed within three.

pre-defined regions of interest using UPD: (1) the full imaging sector (excluding the CG area), (2) cortico-subcortical areas, and (3) basal ganglia within deep gray matter. For each region, arterial and venous CBV were defined as the sum of power Doppler signal intensity in arteries and veins, respectively. Quantification of arterial/venous CBV in cortico-subcortical areas and basal ganglia was only performed within the deep hypothermia cohort. The main interest of this assessment was to examine the impact of deep hypothermia on CAR in these brain regions, which are known to have distinct anatomical and physiological characteristics^10^.

### Statistical analysis

The patients’ demographic and clinical data were compared using Student’s t-test. To account for individual differences, such as unique physiology or the efficiency of CAR, as well as changes occurring between our measurement time points during CPB, we used a linear mixed-effect model to determine the association between arterial/venous CBV and MAP. A Two-Sample t-test was performed to compare: (1) the ratio of arterial/venous CBV changes to MAP changes between patient groups, and (2) the ratio of arterial/venous CBV changes to MAP changes between basal ganglia and cortico-subcortical areas in the “DH” group during DH-CPB. A significant level of 5% was used for all statistical analyses, which were conducted using MATLAB software (Release 2023b, The MathWorks, Inc., Natick, MA, USA).

## Results

In total 12 patients were recruited for the study with 6 patients in the “DH group” and 6 in the “non-DH group”. Table 1 demonstrates the democratic and intraoperative-clinical data. Among all measured parameters, only Intraoperative average MAP showed a statistically significant difference between groups (DH group: 41.82 mmHg, 95% CI: 35.97–47.67; non-DH group: 49.82 mmHg, 95% CI: 48.27–51.36; *p* = 0.016). No significant differences were observed in other parameters, including PCO_2_ (DH group: 33.74 mmHg, 95% CI: 27.78–39.69; non-DH group: 32.15 mmHg, 95% CI: 23.11–41.19; *p* = 0.716) and surgery time (DH group: 350.67 min, 95% CI: 304.84–396.50; non-DH group: 297.83 min, 95% CI: 234.04-361.63; *p* = 0.118).

**Table 1:** Demographic and intraoperative clinical data.

Patient characteristics	Control (*n* = 6)	Deep hypothermia (*n* = 6)	*p*-value
GA (Weeks)	38 (37–39)	39.5 (38.66–40.33)	0.17
Age (Days)	10 (1–20)	3 (2–4)	0.23
Male: Female	3:3	4:2	0.61
Weight, kg	3.18 (2.64–3.60)	3.35 (3.08–3.62)	0.44
BSA, m^2^	0.20 (0.19–0.21)	0.21 (0.20–0.23)	0.12
Head circumference, cm	34.25 (33.26–34.99)	34.58 (33.58–35.57)	0.36
Surgery time, min	297.83 (234.04–361.63)	350.67 (304.84–396.50)	0.11
Intra-operative MAP, mmHg	49.82 (48.27–51.36)	41.82 (35.97–47.67)	0.016
Intra-operative PCO_2,_ mmHg	32.15 (23.11–41.19)	33.74 (27.78–39.69)	0.71

Values are average and (lower and upper 95% confidence intervals). BSA = body surface area; ga = gestational age, map = mean arterial blood pressure; PCO_2_ = partial pressure of carbon dioxide.

### Association between full sector arterial/venous CBV and MAP

**Fig. 2:**
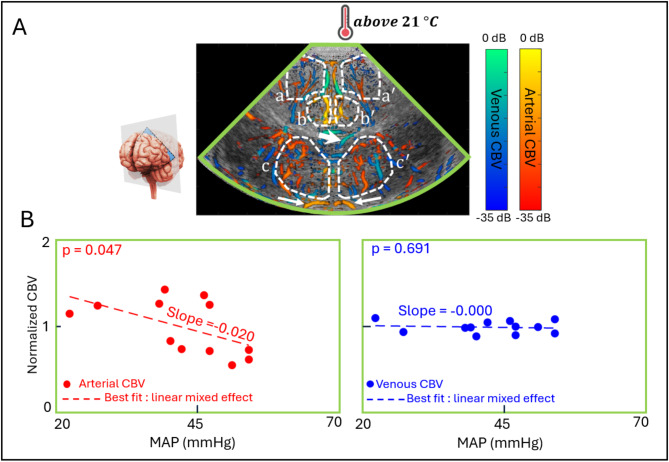
(**A**) Qualitative example of artery/vein segmented power Doppler image superimposed on a B-mode of a single patient in the “non-deep hypothermia group”. Regions a and a’ represent the cortical areas in the right and left hemisphere respectively, b and b’ represent the cingulate gyrus areas in the right and left hemisphere respectively, and c and c’ represent the basal ganglia in the right and left hemisphere respectively. Large arrowhead indicates the subependymal collector vein, and small arrowheads indicate the typical middle cerebral arteries. (**B**) Quantitative results of full sector (excluding the CG area in gray) arterial and venous CBV vs. MAP in the non-deep hypothermia group (*n* = 6). Abbreviations: CBV = cerebral blood volume; CG = cingulate gyrus; DH-CPB = deep hypothermic cardiopulmonary bypass; MAP = mean arterial blood pressure.

**Fig. 3:**
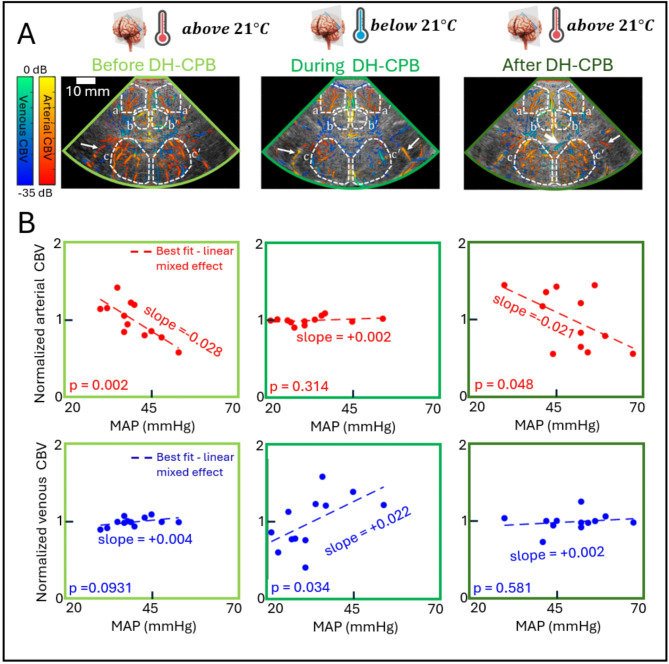
(**A**) Qualitative examples of artery/vein segmented power Doppler image superimposed on a B-mode of a single patient in the “deep hypothermia group” before, during and after DH-CPB. Regions a and a’ represent the cortical areas in the right and left hemisphere respectively, b and b’ represent the cingulate gyrus areas in the right and left hemisphere respectively, and c and c’ represent the basal ganglia in the right and left hemisphere respectively. Large arrowhead indicates the subependymal collector vein, and small arrowheads indicate the typical middle cerebral arteries. (**B**) Quantitative results of full sector (excluding the CG area in gray) arterial and venous CBV vs. MAP in the deep hypothermia group, before (first column), during (middle column), and after (last column) DH-CPB (*n* = 6). Abbreviations: CBV = cerebral blood volume; CG = cingulate gyrus; DH-CPB = deep hypothermic cardiopulmonary bypass; MAP = mean arterial blood pressure.

**Fig. 4:**
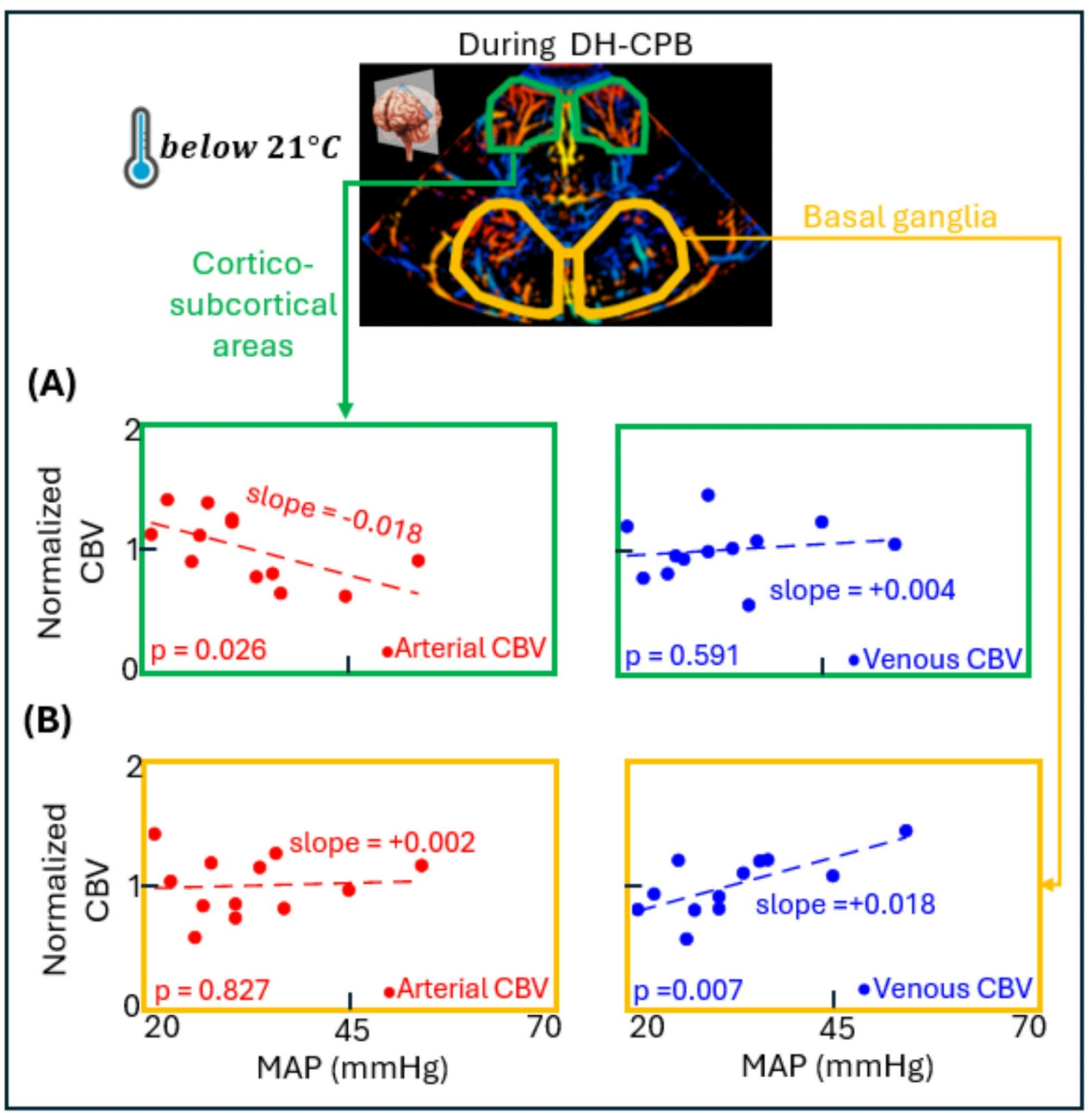
Results of regional arterial and venous CBV vs. MAP in the deep hypothermia group during DH-CPB (n = 6). Panel (**A**) shows these results in cortico-subcortical areas and Panel (**B**) shows these results in the basal ganglia in deep gray matter. Abbreviations: CBV = cerebral blood volume; MAP = mean arterial blood pressure.

Figure 2 shows that in the “non-DH group” there is a significant negative association between arterial CBV and MAP (slope = -0.020 $\:{mmHg}^{-1}$, *p* = 0.047), while there is no significant association between venous CBV and MAP (*p* = 0.691). In the “DH group” similar association patterns to those in the “non-DH group” were observed before and after DH-CPB (See Fig. 3). However, during DH-CPB, no significant association was found between arterial CBV and MAP (*p* = 0.314), whereas a significant positive association was found between venous CBV and MAP (slope = + 0.022 $\:{mmHg}^{-1}$, *p* = 0.034). Here, we would like to clarify few methodological considerations regarding the nature of these results. First, in the deep hypothermia group (Fig. 3), a total of 6 data points is shown for each patient: two before, two during, and two after DH-CPB, as opposed to the total of two data points for each patient in the non-deep hypothermia group (Fig. 2). Second, these data were not analyzed as individual, independent data points. As demonstrated in Figs. 2 and 3, and later in Fig. 4 (Result, Section C), we employed a linear mixed-effect model. Each plot represents 12 data points belonging to 6 patients, clearly showing that the data were analyzed as a whole and not as individual datapoints. This statistical approach, which was also used in our previous publication for repeated measurement during CPB^18^ allows us to capture the complex relationships between measurements and control for individual patient variabilities that are not easily measured..

### Comparisons in full sector between “non-DH group” and “DH group”

No significant differences were found in the ratio of arterial CBV changes to MAP changes between the “non-DH group” and the “DH group” before DH-CPB (*p* = 0.172) and after DH-CPB (*p* = 0.648). However, significant differences were observed during DH-CPB (*p* < 0.001). Similarly, no significant differences were found in the ratio of venous CBV changes to MAP changes before DH-CPB (*p* = 0.104) and after DH-CPB (*p* = 0.799), but significant differences were noted during DH-CPB (*p* = 0.008).

### Association between arterial/venous CBV vs. MAP in cortico-subcortical areas and basal ganglia during DH-CPB (See Fig. 4)

Figure 4A illustrates the association between arterial/venous CBV and MAP in cortico-subcortical areas during DH-CPB, while Fig. 4B shows these associations in basal ganglia. In cortico-subcortical areas, we found a significant negative association between arterial CBV and MAP (slope = -0.018 $\:{mmHg}^{-1}$, *p* = 0.026). In basal ganglia, no significant association was found between arterial CBV and MAP (*p* = 0.827). Conversely, there is a significant positive association between venous CBV and MAP in basal ganglia (slope = + 0.018 $\:{mmHg}^{-1}$, *p* = 0.007), but no significant association in cortico-subcortical areas (*p* = 0.591). Furthermore, significant differences were found between cortico-subcortical areas and basal ganglia for the ratio of arterial CBV changes to MAP changes (*p* = 0.039) and for the ratio of venous CBV changes to MAP changes (*p* = 0.027).

## Discussion

In this study, we combined UPD imaging and artery/vein discrimination to study CBV and CAR in newborns with complex congenital heart disease undergoing cardiac surgery requiring cardiopulmonary bypass. We investigated two groups: patients undergoing cardiopulmonary bypass with deep hypothermia (*n* = 6, “DH group”) and patients undergoing cardiopulmonary bypass without deep hypothermia (*n* = 6, “non-DH group”). Our findings reveal that: (A) in the “non-DH group”, there is a significant negative association between full sector arterial CBV and MAP, while no significant association exists between full sector venous CBV and MAP; (B) during DH-CPB, there is no significant association between full sector arterial CBV and MAP, but a significant positive association exists between full sector venous CBV and MAP; (C) significant differences in the ratio of arterial/venous CBV to MAP changes are observed across deep and cortical brain regions during DH-CPB.

### Observations from the non-DH group: an insight on the cerebral physiology

**Fig. 5:**
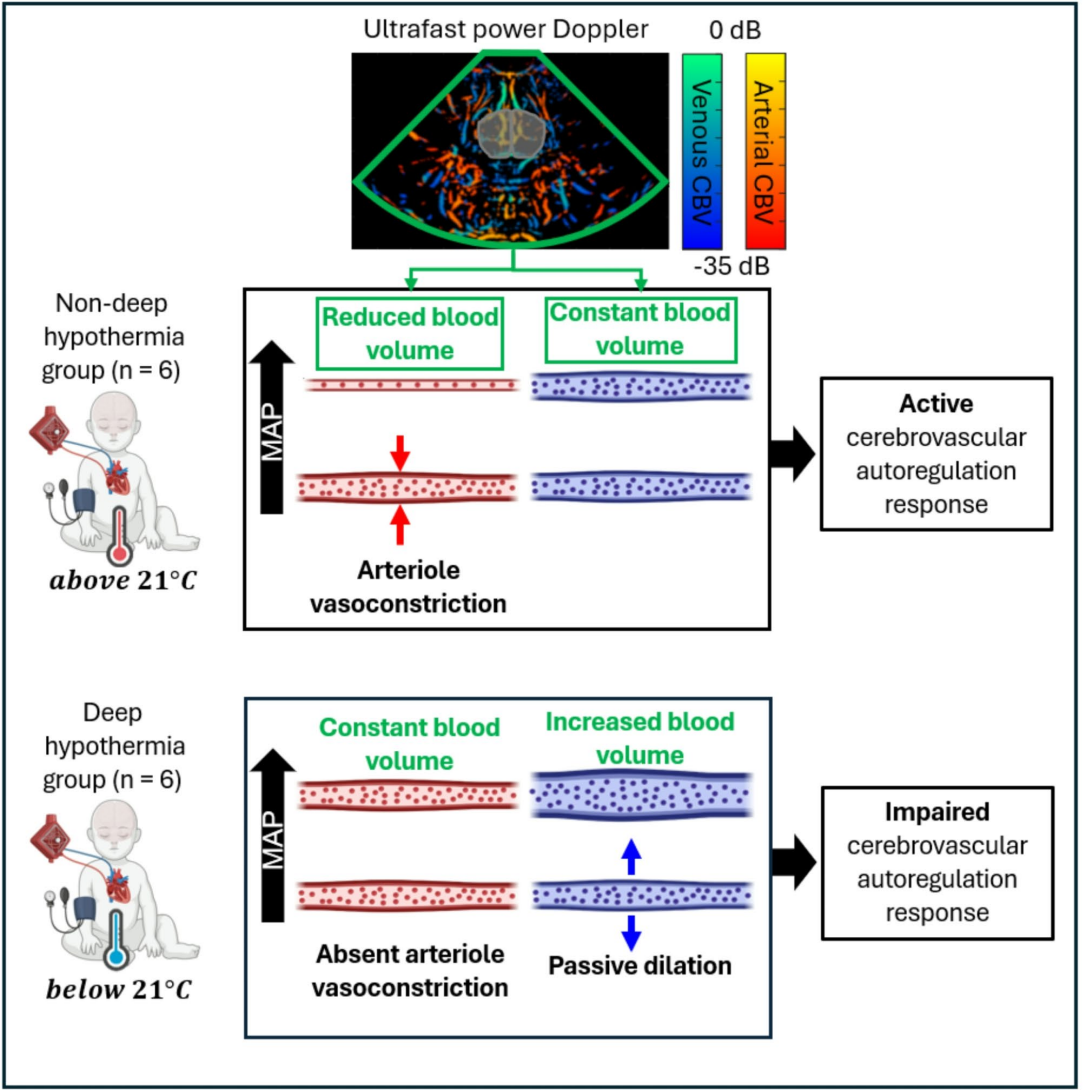
Interpretation of results in the full imaging sector of ultrafast power Doppler for non-deep hypothermia group (top panel), and deep hypothermia group (bottom panel). Abbreviations: CBV = cerebral blood volume; MAP = mean arterial blood pressure.

Regarding the “non-DH” group results (Fig. 2), we observed a significant negative association between full sector arterial CBV and MAP. Given that CAR remains intact for this group, this pattern must be interpreted within this specific context. When MAP increases, the arterial diameter is expected to decrease to maintain a constant cerebral blood flow. This decrease in arterial diameter leads to a decrease in arterial CBV, as shown in Fig. 5. This response aligns with the known principles of cerebral autoregulation, where arterioles constrict in response to elevated pressure^7^. Bouma et al.^20^ previously demonstrated that cerebral arterioles exhibit an exponential decrease in diameter in response to acute hypertension, a mechanism designed to prevent excessive capillary pressure and reduce the risk of brain injury. Therefore, our findings suggest that CAR is preserved in the “non-DH group”. Consistent with our findings, Taylor et al.^31^ previously showed that CAR is active in infants and neonates during normothermic CPB. In their study, transcranial ultrasound Doppler was used to study the cerebral pressure-flow velocity relationship in the M1 segment of the middle cerebral arteries in 25 neonates and infants ranging from 3 to 210 days of age. Similar observations were made by Greeley et al.^32^ in the context of neonates and infants undergoing moderate hypothermia CPB. The authors showed that cerebral vasculature maintains a normal physiologic response of constriction during high perfusion pressure and dilation when perfusion pressure is low. Considering that CAR is active for this “non-DH group” the absence of association between full sector venous CBV and MAP is not unexpected. Under normal physiological conditions, systemic blood pressure decreases rapidly across the resistance arteries and arterioles, reaching low values in the capillaries and venous vessels (approximately 4–8 mmHg)^33^. Fluctuations in MAP should result in minimal changes in venous pressure and, therefore, not significantly impact venous CBV.

### Impact of deep hypothermia on CAR

Contrary to the “non-DH” group, we observed that the association between full sector arterial CBV and MAP was not significant during DH-CPB in the “DH” group. This absence of variation in arterial CBV is likely due to the absence of arterial vasoconstriction or vasodilation, indicating impaired CAR response (See Fig. 5, bottom panel). Previous studies have suggested that severe temperature reductions during cardiac surgery impair vascular relaxation and its ability to vasoconstrict or vasodilate in response to altered perfusion pressure^35–37^. This implies that temperature reduction below 21 °C is attributed to the loss of vascular tone. Moreover, Smith et al.^37^ recently demonstrated that induced deep hypothermia in neonatal CPB is associated with hypotension, increased cerebral oxygen saturation measured by NIRS, and dysautoregulation of the cerebrovascular network. Based on a review paper on neonates undergoing cardiac surgery, it was noted that such alterations in vascular behaviour during DH-CPB are likely caused by cold-induced cerebrovasoparesis^38^.

During DH-CPB, we found a significant positive association between full sector venous CBV and MAP (See Fig. 3). This relationship is not observed under normal physiological conditions and is consistent with impaired CAR during DH-CPB. Without active regulation of vascular tone, changes in MAP will cause higher pressures to be transmitted downstream to capillaries, and ultimately the venous system, resulting in venous dilatation and an increase in venous CBV with increasing MAP. While we are not aware of an equivalent examination of venous CBV, a study examining total liquid ventilation treatment in a rabbit model found a positive association between total CBV and MAP under these conditions, consistent with loss of CAR^39^. It is important to note that most of the CBV signal arises from veins due to their larger lumen diameter and higher compliance in terms of pressure-volume relationship. This explains why the expected positive association during impaired autoregulation is primarily manifested in our venous CBV measurements, while no significant association was found with arterial CBV.

Another key finding of this paper is the observed ratio of changes in the full-sector arterial/venous CBV to the change in MAP before and after DH-CPB. We found that these ratios did not significantly differ from those in the “non-DH” group but were significantly different when compared to the same population group but during DH-CPB. Therefore, we concluded that temperature management strategies during CPB are essential for preserving CAR and maintaining stable hemodynamics in neonates with complex congenital heart disease undergoing CPB.

### Adaptation of local brain perfusion: difference between cortex and deep structures

Finally, we observed significant arterial/venous CBV variation differences between cortico-subcortical areas and basal ganglia during DH-CPB. Such heterogeneity in blood volume variations may indicate the region-specific nature of CAR, which could vary depending on the regions analyzed. Aguet et al.^18^ showed differences in full CBV variations between regional cortical, deep grey matter or large vessel CBV, suggesting physiological differences in CAR depending on the vessel calibre and the cerebral region. Furthermore, Steventon et al.^40^ and Warnert et al.^41^ have previously demonstrated varying responses to hypoxic stress between large central and peripheral arteries, emphasizing the critical role of the central arteries of the circle of Willis in regulating cerebral vascular resistance. In neonates with hypoxic-ischemic encephalopathy, insufficient blood flow and perinatal asphyxia lead to loss of normal CAR resulting in brain injury^42,43^. Regional differences in cerebral blood flow adaptation were reported in these patients, suggesting regional vulnerability to compromised perfusion^44^. Physiologically, it is important to consider variations in metabolic demand and brain activity throughout the analyzed regions. For example, the basal ganglia in the deep gray matter are more metabolically active compared to cortico-subcortical areas in neonates, making them more vulnerable to hypoxic-ischemic injury^45^. Bearing in mind that the desired effect of deep hypothermia is myocardial and cerebral protection by reducing metabolic activity^47,47^, the observed regional variations in CAR response during DH-CPB are not unexpected.

### Study limitations

Our study has several limitations. First, we did not account for the influence of anesthetic agents on cerebrovascular physiology, in particular, the vasodilatory effect of isoflurane, a commonly used anesthetic gas^48^. Second, this was a pilot study conducted on a small sample size for both patient cohorts (*n* = 6). Therefore, it is challenging to extrapolate these results to a general population. Although our study was not conducted on a large population set, we were able to identify important differences between the study groups. One limitation of the study design is that the measurement of relationship between MAP and CBV relies on consistency between measurements of CBV at different stages of the surgical procedure. This is made difficult by the nature of power Doppler ultrasound where the signal is dependent on the geometrical factors of the ultrasound beam and attenuation in addition to blood volume. In the present study these confounding factors are minimized by consistent placement of the ultrasound transducer between scans. Attenuation compensation for fractional moving blood volume measurements has been described by Zhang et al.^49^ and could be a potential solution, although validating this technique with phased array probes and diverging waves in neonatal brain imaging requires further exploration. For the current study, considering that we were mainly interested in the longitudinal variation of CAR, this lack of absolute measurements for arterial/venous CBV is not a major limitation. Another limitation of our study was that we did not account for the effects of PaCO_2_ on CAR. Indeed, it is well known that PaCO_2_ is a potent vasodilator and could significantly affect cerebral blood flow and CBV^50^. While we monitored PaCO_2_ intraoperatively, its vasodilatory effects cannot be easily isolated from other factors affecting vessel tone during CPB, such as anesthetics and nitric oxide used during surgery^18^. This was also another reason that a linear mixed-effect model was used in this study, considering the multifactorial nature of vascular tone regulation during cardiac surgery and the potential individual variability among patients^51^. Despite PaCO_2_ being a potent vasodilator, an increase during deep hypothermia, when arterial vessels are maximally dilated due to vasoplegia, is unlikely to impact arterial CBV. However, future studies should aim to systematically analyze the specific contribution of PaCO_2_ to arterial/venous CBV changes during CPB. Lastly, considering the linear model which was used in our study to establish the association between CBV and MAP, we would like to clarify that this relationship is more complex and often follow a sigmoidal curve^52^. In that regard, future research should employ more sophisticated statistical approaches to more accurately characterize the complex, non-linear relationship between MAP and CBV.

### Perspective

In this study, we have demonstrated the high sensitivity of ultrafast ultrasound imaging to assess.

CAR in neonates with complex congenital diseases. In contrast to NIRS, ultrafast ultrasound imaging could be used to quantify CAR in the whole brain and in specific cerebral regions. The high spatiotemporal coherence of ultrafast ultrasound imaging combined with angle-independent power Doppler measurements overcomes the key limitations of transcranial Doppler ultrasound, enabling both reliable quantification and detection of small arterioles (250 μm) in which CAR is primarily more dominant. Moreover, the use of advanced clutter filtering techniques (e.g., SVD) in ultrafast ultrasound imaging effectively suppresses motion artifacts that may compromise measurements in other monitoring approaches such as non-invasive ICP monitoring and laser Doppler flowmetry. These advantages over the existing techniques may allow for a better understanding of CAR during DH-CPB. This preliminary work focused on the technical aspects of the method. Future clinical studies aiming to use ultrafast ultrasound imaging as a continuous monitoring technique to assess CAR during DH-CPB are needed. From a clinical standpoint, improving our understanding of autoregulatory thresholds is crucial, as the normal cerebral autoregulatory range for neonates and children is not very well understood^53^. Current clinical practice during CPB in this population typically maintains MAP between 30 and 50 mmHg^54^. However, in a study performed by Brady et al.^55^, it was found that the lower limit of CAR was approximately 42 mmHg during cardiac surgery, suggesting potential variability in these thresholds. It is important to note that individual infant autoregulatory thresholds may shift during evolving brain injury and medical interventions. For instance, whole-body hypothermia following hypoxia can decrease the lower limit of the pressure-flow curve, although this represents an acute response that is not sustained with prolonged hypothermia^56^. Moreover, in a piglet model of hydrocephalus, researchers demonstrated that the lower limit of CAR shifted to a higher perfusion pressure during intracranial hypertension from cephalic venous outflow obstruction with intracranial venous congestion^57^. Pesek et al.^58^ also showed that intracranial hypertension can move the upper limit of CAR to a lower perfusion pressure. These combinations of variations in the lower and upper limits could potentially cause the autoregulatory plateau to narrow. While determining these precise autoregulatory limits was not within our current scope, future studies using our proposed technique may provide deeper insights into these critical thresholds. The use of 3D ultrafast ultrasound imaging could also offer new insights into brain perfusion by enabling (1) the measurement of cerebral blood flow autoregulation or vascular remodelling after ischemic stroke recovery^59^ and (2) the visualization of large areas of the brain, potentially reducing operator dependency and improving the detection of scattered lesions, thereby mitigating the risk of CAR impairment^60,61^.

## Conclusion

In this study, we used UPD combined with artery and vein discrimination to assess CAR in neonates undergoing cardiopulmonary bypass surgery, with and without deep hypothermia. We observed that CBV varies according to 3 main parameters: (1) the state of CAR (active or impaired); (2) the vascular sector analyzed (arteries or veins); and (3) the cerebral region (cortical versus deep regions). From a clinical perspective, our findings emphasize the critical importance of temperature management during cardiopulmonary bypass. Our study paves the way for further exploration and validation of continuous monitoring of CAR by UPD to optimize neurovascular management strategies during CPB in this vulnerable patient population.

## Data Availability

Data are available from the corresponding authors upon reasonable request and with permission from the Hospital for Sick Children.

## Abbreviations

CAR: Cerebrovascular autoregulation
DH-CPB: Deep hypothermia cardiopulmonary bypass
CBV: Cerebral blood volume
UPD: Ultrafast power Doppler
NIRS: Near-Infrared spectroscopy
MAP: Mean arterial blood pressure
AUC: Area under the curve
NDS: Normalized ultrafast Doppler spectrogram
CG: Cingulate gyrus
